# Supplementary material for: AZD5582 robustly reactivates latently infected cells and clears the majority of those reactivated from the SIV reservoir
Source: bioRxiv. 2026 Jun 2:2026.06.01.729198. Preprint. [Version 1] doi: 10.64898/2026.06.01.729198 (PMC13252412; doi:10.64898/2026.06.01.729198)
Supplement: Supplement 1 [file NIHPP2026.06.01.729198v1-supplement-1.pdf]

bioRxiv preprint doi: https://doi.org/10.64898/2026.06.01.729198; this version posted June 2, 2026. The copyright holder for this preprint (which was not certified by peer review) is the author/funder. This article is a US Government work. It is not subject to copyright under 17 USC 105 and is also made available for use under a CC0 license.

Supplementary Materials

S1 Text. Modeling Plasma AZD concentration.

We fit a one-compartment PK model to plasma AZD concentration

$$Z' = k_a D(t) - kZ$$

$$D(t) = \begin{cases} 1, & t_n \le t < t_n + \Delta_t \\ 0, & t \ge t_n + \Delta_t \end{cases}.$$

$Z(t)$ is plasma AZD concentration, k_a is the infusion rate constant over the infusion duration Δ_t, and k is the tissue distribution rate constant. The function $D(t)$ represents the dosing of AZD at time t_n. The plasma AZD concentration data, reported in unit of ng/mL, comes from Nixon et al. [30]. However, Sampey et al. reported EC50 for AZD in unit of nM [31], so we convert the unit to nM using AZD molecular weight of 1015.29 g/mol [76]. Model fitting is carried out with MATLAB built-in function *fmincon* using 10 random initial guesses with the function *MultiStart*. Model fit is shown in Fig. S1, and best-fit parameters are presented in Table S1.

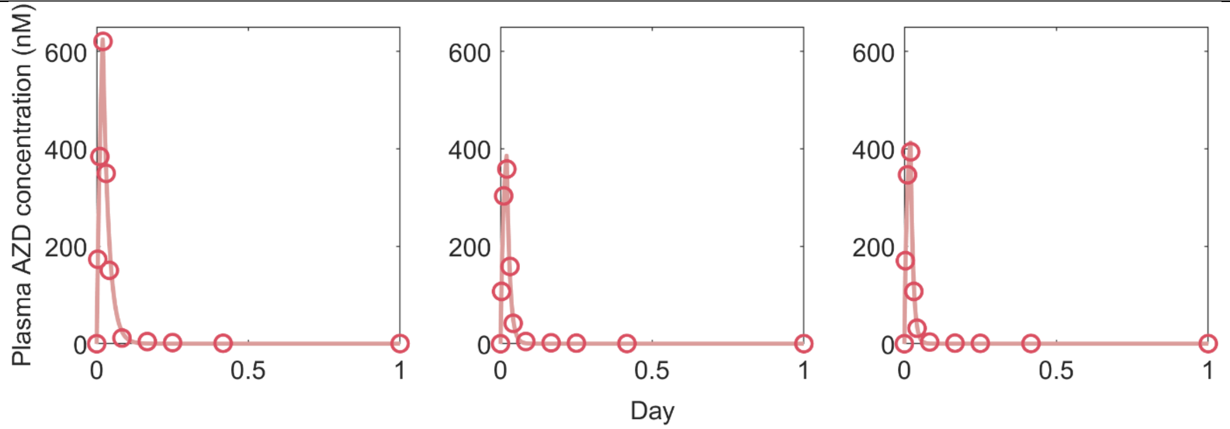

Figure S1. One compartment PK model fit. Red curves are model fit. Open circles are plasma AZD data from three macaques [30].

ID	1	2	3
k_a (day^{-1})	5.50×10^4	4.17×10^4	6.10×10^4
k (day^{-1})	68.92	86.81	131.21
Δ_t (day)	1/48	1/48	1/48

Table S1. Estimated PK parameters for AZD.

S2 Text. Mathematical Models M1 – M31

We examined a family of models resulting from the modeling framework introduced by Conway and Perelson [9], which has been shown to accurately capture the HIV and SIV viral load trajectory under different scenarios [11,61–63]. The general model follows Policicchio et al. [61] and Cao et al. [62], where the Conway-Perelson model is extended to include pre-integration

bioRxiv preprint doi: https://doi.org/10.64898/2026.06.01.729198; this version posted June 2, 2026. The copyright holder for this preprint (which was not certified by peer review) is the author/funder. This article is a US Government work. It is not subject to copyright under 17 USC 105 and is also made available for use under a CC0 license.

infected cells and an exhausted state of effector cells are incorporated (Fig. 2A). Note that the ART regimes in the three groups contain DTG, an integrase inhibitor, necessitating the need for a separation of pre-integrated long-lived and short-lived infected cells [61]. We focused on the period when the SIV-infected macaques were under ART and treated with AZD, so we exclude effector cell exhaustion and details of the infection process (gray boxes in Fig. 2A). The remaining components include productively infected cells I, latently infected cells L, and effector cells E, which make up the basic model (Fig. 2A). The four main variations of the baseline model that we consider are presented in Fig. 2B-E. We present the equations for 31 model iterations (M1 – M31). Parameter definitions and ranges are shown in Table S2. Model fit comparison is presented in Fig. S3 and Table S3.

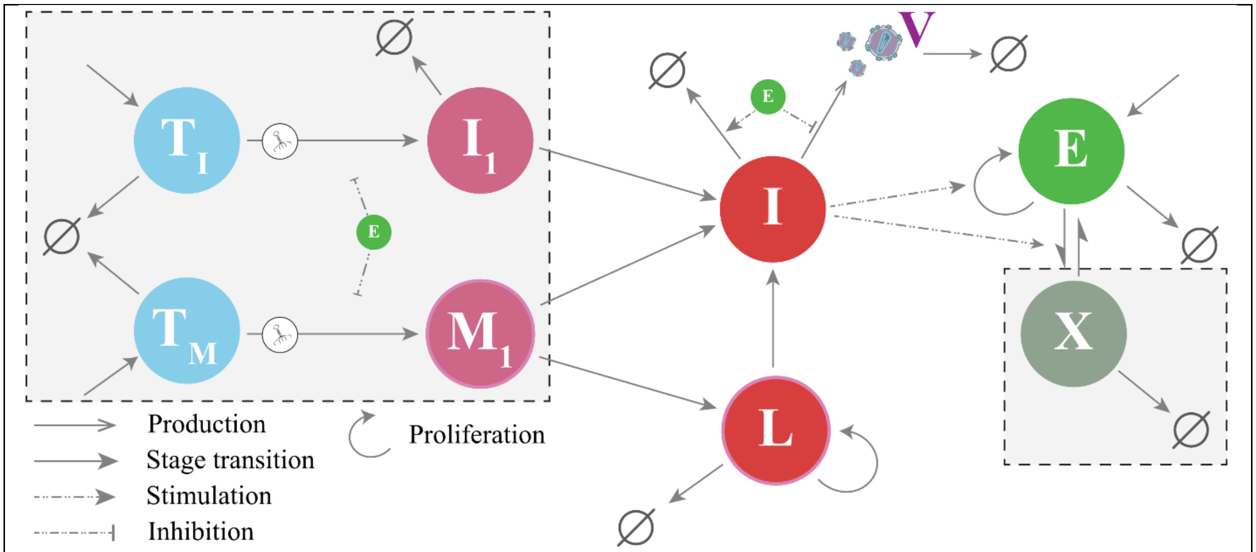

Figure S2. Baseline viral dynamic model. Viruses (V) enter short- (T_I) and long-lived (T_M) target cells creating pre-integrated infected short- and long-lived cells I_1 and M_1, respectively. After viral integration, these cells become productively infected cells (I), with a fraction of the long-lived cells entering latency (L). Effector cells (E) expand in response to the infection and becomes exhausted (X) over time. During ART, the elements outside of the gray boxes are sufficient to describe viral dynamics.

M1.

$$L_0' = -d_L L_0 + \rho L_0 - a L_0 - \frac{\alpha Z(t)}{EC_{50} + Z(t)} L_0 + f_L \beta_V V$$

$$I' = a L_0 + \frac{\alpha Z}{EC_{50} + Z} L_0 - \delta_I I - m_1 E I + (1 - f_L) \beta_V V$$

$$V' = \frac{pI}{1 + m_2 E} - cV$$

$$E' = \lambda_E - d_E(t) + \frac{bEI}{K_B + I}$$

M2.

bioRxiv preprint doi: https://doi.org/10.64898/2026.06.01.729198; this version posted June 2, 2026. The copyright holder for this preprint (which was not certified by peer review) is the author/funder. This article is a US Government work. It is not subject to copyright under 17 USC 105 and is also made available for use under a CC0 license.

$$L_R' = -d_L L_R + \rho L_R - aL_R + (1 - f_S) f_L \beta_V V$$
$$L_S' = -d_L L_S + \rho L_S - aL_S - \frac{\alpha Z(t)}{EC_{50} + Z(t)} L_S + f_S f_L \beta_V V$$
$$I' = a(L_R + L_S) + \frac{\alpha Z}{EC_{50} + Z} L_S - \delta_I I - m_1 EI + (1 - f_L)\beta_V V$$
$$V' = \frac{pI}{1 + m_2 E} - cV$$
$$E' = \lambda_E - d_E(t) + \frac{bEI}{K_B + I}$$

M3.

$$L_0' = -d_L L_0 + \rho L_0 - aL_0 - \frac{\alpha Z(t)}{EC_{50} + Z(t)} L_0$$
$$A' = \frac{\alpha Z}{EC_{50} + Z} L_0 - \epsilon_A \delta_I A - \epsilon_A m_1 EA$$
$$I' = aL_0 - \delta_I I - m_1 EI$$
$$V' = \frac{p(I + \epsilon_A A)}{1 + m_2 E} - cV$$
$$E' = \lambda_E - d_E(t) + \frac{bE(\epsilon_A A + I)}{K_B + \epsilon_A A + I}$$

M4. We show N=M=3 as the initial demonstration for this variation.

$$L_0' = -d_L L_0 - aL_0 + \rho L_0 - \frac{\alpha Z(t)}{EC_{50} + Z(t)} L_0 + \omega R_3$$
$$L_i' = \gamma(L_{i-1} - L_i), \quad i = 1, \dots, 3$$
$$A' = \gamma L_3 - \omega A - \epsilon_A \delta_I A - \epsilon_A m_1 EA$$
$$R_1' = \omega(A - R_1)$$
$$R_j' = \omega\left(R_{j-1} - R_j\right), \quad j = 2, \dots, 3$$
$$I' = aL - \delta_I I - m_1 EI$$
$$V' = \frac{p(I + \epsilon_A A)}{1 + m_2 E} - cV$$
$$E' = \lambda_E - d_E(t)E + bE \frac{\epsilon_A A + I}{K_B + \epsilon_A A + I}$$

M5.

$$L_R' = -d_L L_R - aL_R + \rho L_R$$
$$L_S' = -d_L L_S + \rho L_S - aL_S - \frac{\alpha Z(t)}{EC_{50} + Z(t)} L_S + \omega R_3$$
$$L_1' = \gamma(L_S - L_1)$$
$$L_i' = \gamma(L_{i-1} - L_i), \quad i = 2,3$$
$$A' = \gamma L_3 - \omega A - \epsilon_A \delta_I A - \epsilon_A m_1 EA$$
$$R_1' = \omega(A - R_1)$$

bioRxiv preprint doi: https://doi.org/10.64898/2026.06.01.729198; this version posted June 2, 2026. The copyright holder for this preprint (which was not certified by peer review) is the author/funder. This article is a US Government work. It is not subject to copyright under 17 USC 105 and is also made available for use under a CC0 license.

$$R_j' = \omega(R_{j-1} - R_j), \;\; j = 2, \dots, 3$$
$$I' = a(L_R + L_S) - \delta_I I - m_1 E I$$
$$V' = \frac{p(I + \epsilon_A A)}{1 + m_2 E} - cV$$
$$E' = \lambda_E - d_E(t)E + bE\frac{\epsilon_A A + I}{K_B + \epsilon_A A + I}$$

M6 – M10 as M1 – M5 but without an effector compartment.

M11 – M12 as M6 – M7 but without active infection ($\beta_V = 0$).

M13 – M15 as M3 – M5 but with active infection.

M16 – M18 as M13 – M15 but without an effector compartment, or equivalently, as M8 – M10 with active infection.

M19 – M22 as M9 but with $L_0(0)$ fixed to 10 (M19), 1 (M20), 0.1 (M21) cell per mL, or fitted without random effect (M22).

M23 – M27 as M22 but with N=M=5 (M23), =7 (M24), =9 (M25), =10 (M26 – lowest BICc), =11 (M27).

M28 as M26 but without refractory state – AZD-reactivated cells return directly to being susceptible for further induction.

M29 as M26 but with ϵ_A separated into ϵ_A^i, for the effect of infected cell killing and viral production.

M30 as M10 but with the same modifications as M26.

M31 as M10 but with the same modifications as M29 (lowest -2LL).

bioRxiv preprint doi: https://doi.org/10.64898/2026.06.01.729198; this version posted June 2, 2026. The copyright holder for this preprint (which was not certified by peer review) is the author/funder. This article is a US Government work. It is not subject to copyright under 17 USC 105 and is also made available for use under a CC0 license.

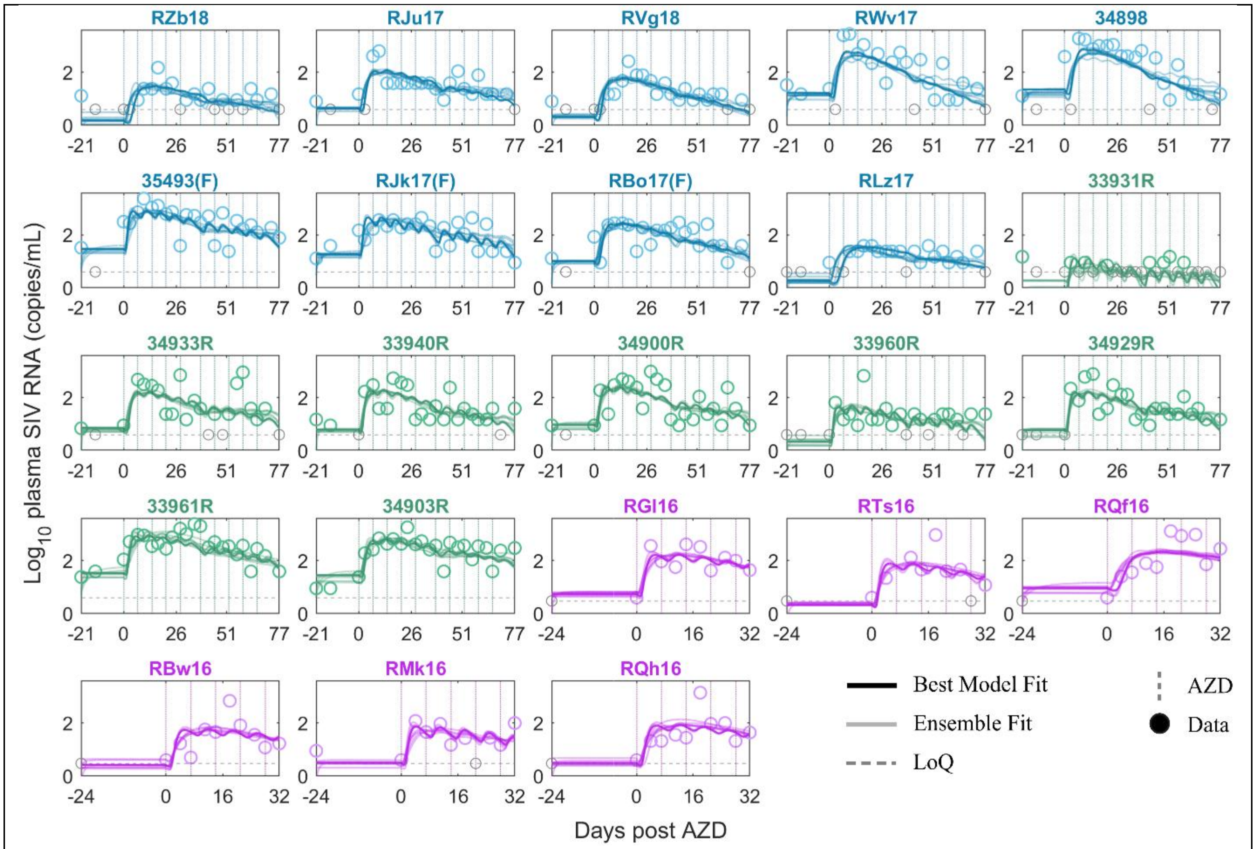

Figure S3. Ensemble and best model fit. Best model (M26) (bolded solid curve). Blue is the group treated with AZD, N-803, and RhmAbs. Green is the group treated with AZD and RhmAbs. Purple is the group treated with anti-CD8α prior to AZD. Ensemble model fit is represented by the transparent curves. Gray circles are data points below the limit of quantification (dotted horizontal gray line). Non-gray circles are data points above the limit of quantification. The dotted vertical lines show AZD administrations.

Parameter	*Definition & Unit*	*Range/value*	*Ref*
$log_{10}\, p$	Viral production rate (day^{-1})	$3 - 5$	[9,11]
$L_0(0)$	Initial size of latent reservoir (cell mL^{-1})	$0.01 - 10$	[96,97]
m_1	Effector cell killing rate (day^{-1})	$0.001 - 10$	[9,11]
m_2	Effector cell reduction of viral production (cell^{-1})	$0.001 - 10$	-
m_3	Effector cell reduction of viral infection (cell^{-1})	$0.001 - 10$	-
b	Maximum effector cell expansion rate (day^{-1})	$0.1 - 10$	[9,11]
K_B	Density of infected cells needed to reach a half-maximal effector cell expansion rate (mL^{-1})	$0.1 - 10000$	[11,98]
α	Maximum reactivation rate of the noncanonical NF-κB pathway by AZD (day^{-1})	$0.01 - 100$	-
γ	Activation transition rate (day^{-1})	$0.1 - 10$	-
ω	Refractory transition rate (day^{-1})	$0.01 - 10$	-
f_S	Fraction of the latent reservoir susceptible to activation by AZD (unitless)	$0.01 - 1$	-

bioRxiv preprint doi: https://doi.org/10.64898/2026.06.01.729198; this version posted June 2, 2026. The copyright holder for this preprint (which was not certified by peer review) is the author/funder. This article is a US Government work. It is not subject to copyright under 17 USC 105 and is also made available for use under a CC0 license.

$log_{10}\,\beta_V$	Infection rate constant (viral copies^{-1} day^{-1})	$3\,-\,5$	-
ϵ_A	Relative reactivation efficiency ratio (unitless)	$0.01\,-\,1$	-
ϵ_A^1	ϵ_A - specific to cytotoxicity (unitless)	$0.01\,-\,1$	-
ϵ_A^2	ϵ_A - specific to viral production (unitless)	$0.01\,-\,1$	-
ϵ_A^3	ϵ_A - specific to the susceptibility to effector cell cytolytic effect (unitless)	$0.01\,-\,1$	-
ϵ_A^4	ϵ_A - specific to stimulation of effector cell expansion (unitless)	$0.01\,-\,1$	-
$log_{10}\,\zeta$	Scaling constant to CA-DNA data (unitless)	$1\,-\,3$	-
k_a	Infusion rate constant (day^{-1})	5×10^4	*
k	tissue distribution rate constant (day^{-1})	90	*
Δ_t	Infusion duration (day)	1/48	*
EC_{50}	Concentration of AZD required to reach half-maximal initiation rate (nM)	7.5	[31]
a	Reactivation rate of latently infected cells (day^{-1})	0.001	[9]
d_L	Death rate of the latent reservoir (day^{-1})	0.004	[64,99]
ρ	Latently infected cell proliferation rate (day^{-1})	$a + d_L - \frac{\ln 2}{t_{1/2}}$	[9,46]
c	Viral clearance rate (day^{-1})	23	[100]
δ_I	Death rate of productively infected cells (day^{-1})	1	[71,101]
λ_E	Production rate of effector cells (day^{-1})	1	[9]
d_E	Death rate of effector cells (day^{-1})	2	[102]

Table S2. Parameter definition. In the Reference column, asterisk indicates fixed values from fitting to data of AZD concentration in blood, and dashed line represents ad hoc ranges. For example, ϵ_A is bounded between 0.01 and 1 to ensure AZD-reactivated cells do not produce more viruses than productively infected cells. The ranges for m_2 and m_3 follow that of m_1. The maximum reactivation rate α is assumed to be at least greater than a, but no more than 100 per day. Previous estimates [10,103] of the natural reactivation rate without ART are higher than the value used here; however, simulations using M26 with a in a similar range (up to 0.1 per day) do not show qualitative differences once AZD begins (except for the steady state value prior to AZD). This reflects that natural reactivation (which is separated from AZD-induced reactivation in our model formulation) is not sufficient to sustain VL during ART. Lastly, note that the estimated parameter values are away from the boundary of these ranges.

**Model**	**-2LL**	**BICc**	**Variation**	**Effector compartment**	**Active infection**	**Refractory State**	**Resistant subpopulation**
M26	851.8	**933.1**	3	No	No	Yes	No
M25	852.3	933.7	3	No	No	Yes	No
M27	852.5	933.8	3	No	No	Yes	No
M24	853.8	935.1	3	No	No	Yes	No
M29	844.7	935.4	3	No	No	Yes	No
M23	857.1	938.5	3	No	No	Yes	No
M19	864.7	939.7	3	No	No	Yes	No
M30	851.3	942.0	4	No	No	Yes	Yes
M31	**842.3**	942.4	4	No	No	Yes	Yes

bioRxiv preprint doi: https://doi.org/10.64898/2026.06.01.729198; this version posted June 2, 2026. The copyright holder for this preprint (which was not certified by peer review) is the author/funder. This article is a US Government work. It is not subject to copyright under 17 USC 105 and is also made available for use under a CC0 license.

M22	864.1	945.4	3	No	No	Yes	No
M9	865.1	949.5	3	No	No	Yes	No
M10	859.4	953.3	4	No	No	Yes	Yes
M28	878.6	959.9	3	No	No	Yes	No
M17	866.7	960.6	3	No	Yes	Yes	No
M18	861.1	964.3	4	No	Yes	Yes	No
M16	901.6	976.6	3	No	No	No	No
M8	918.2	983.9	3	No	No	No	No
M7	910.7	985.8	2	No	Yes	No	Yes
M4	865.9	987.9	3	Yes	No	Yes	No
M5	862.3	993.7	4	Yes	No	Yes	Yes
M6	929.2	994.9	1	No	Yes	No	No
M14	870.2	1011.0	3	Yes	Yes	Yes	No
M3	910.5	1013.7	3	Yes	No	No	No
M15	867.8	1017.9	4	Yes	Yes	Yes	No
M13	903.2	1025.2	3	Yes	No	No	No
M2	913.2	1025.8	2	Yes	Yes	No	Yes
M1	930.1	1033.3	1	Yes	Yes	No	No
M20	1033.6	1108.7	3	No	No	Yes	No
M11	1102.8	1159.1	1	No	No	No	No
M12	1103.7	1169.4	2	No	No	No	No
M21	1569.8	1644.9	3	No	No	Yes	No

Table S3. Model fitting comparison order by increasing BICc. Variation refers to the four basic structures in Fig. 2B-D (main text). The "Yes" or "No" indicates the presence (Yes) or absence (No) of an effector compartment, active infection, refractory state, and resistant subpopulation within the model. Shaded rows are the 10 selected models to form the model ensemble. Bolded values are the smallest -2LL and BICc. Note: M23, M24, M25, M27 share the same mechanism as M26, so we omit them from the model ensemble. M19 is the same as M22 and has a lower BICc; however, this is because the fixed value of $L_0(0)$ for M19 happens to be similar to the value estimated for M22. The value of $L_0(0)$ is important, as demonstrated by the difference in BICc across the three models with different fixed values (M19, 20, 21). Thus, we select the model that allows for the possibility of estimating this value for the model ensemble.

	M9	M10	M17	M18	M22	M26	M28	M29	M30	M31
$\log_{10} p$	4.33	4.22	4.03	4.28	4.21	4.23	4.11	4.07	4.25	4.10
$L_0(0)$	8.32	9.89	8.97	5.17	10.0	9.96	9.99	9.99	9.99	9.99
α	7.54	6.01	12.54	82.67	8.89	4.56	3.60	4.79	4.89	4.92
γ	0.44	0.37	0.46	0.19	0.40	2.25	2.14	2.28	2.26	2.34
ω	0.055	0.028	0.052	0.230	0.058	0.040	3e-8	0.082	0.048	0.062
f_S	-	0.86	-	-	-	-	-	-	0.99	0.99
$\log_{10} \beta_V$	-	-	3.02	4.01	-	-	-	-	-	-
ϵ_A	0.13	0.30	0.07	0.68	0.11	0.16	0.25	-	0.15	-
ϵ_A^1	-	-	-	-	-	-	-	0.15	-	0.13
ϵ_A^2	-	-	-	-	-	-	-	0.24	-	0.23

bioRxiv preprint doi: https://doi.org/10.64898/2026.06.01.729198; this version posted June 2, 2026. The copyright holder for this preprint (which was not certified by peer review) is the author/funder. This article is a US Government work. It is not subject to copyright under 17 USC 105 and is also made available for use under a CC0 license.

$\boldsymbol{log_{10}\,\zeta_1}$	1.49	1.37	1.39	1.67	1.24	1.28	1.26	1.24	1.30	1.24
$\boldsymbol{log_{10}\,\zeta_2}$	1.81	1.70	1.70	1.95	1.69	1.65	1.83	1.56	1.60	1.54
$\boldsymbol{log_{10}\,\zeta_3}$	1.58	1.44	1.41	1.62	1.43	1.45	1.61	1.36	1.38	1.34

Table S4. Population parameter estimates of the model ensemble. Dash indicates the model does not contain the parameter. ξ_1 corresponds to the group treated with AZD, N-803, and RhmAbs. ξ_2 corresponds to the group treated with AZD and RhmAbs. ξ_3 corresponds to the group treated with anti-CD8α prior to AZD.
